# Economic burden of malaria in children under the age of five in Burundi

**DOI:** 10.1186/s12936-025-05684-0

**Published:** 2025-12-01

**Authors:** Fulgence Niyibitegeka, Audace Manirakiza, Nina Hezagira, Pierre Sinarinzi, Patrick Abraham, Angela Devine

**Affiliations:** 1 Economics of Global Health and Infectious Diseases Unit, Centre for Health Policy, Melbourne School of Population and Global Health, University of Melbourne, 207 Bouverie St, Carlton VIC, Melbourne, 3053 Australia; 2 Independent Researcher, Bujumbura, Burundi; 3 National Malaria Control Programme, Ministry of Public Health, Bujumbura, Burundi; 4 Division of Global and Tropical Health, Menzies School of Health Research, Charles Darwin University, Darwin, Australia

**Keywords:** Malaria, Cost-of-illness, Economic burden, Healthcare costs, Direct costs, Indirect costs, Household costs, Productivity loss

## Abstract

**Background:**

Although malaria is the leading killer of children under the age of five in Burundi, limited evidence on the associated costs are available. This study estimates the cost per malaria episode from the household, health system, and societal perspectives for children under the age of five in Burundi.

**Methods:**

A micro-costing approach was used to estimate the cost per episode at community, primary, secondary and tertiary levels of the healthcare system at 15 sites across all five geographic regions in Burundi. Household costs were collected through interviews with caregivers using a structured questionnaire, while health system costs were determined by review of patient medical and financial records.

**Results:**

The average societal cost per episode was US$12.1. Households shouldered most of these costs, with US$3.3 in out-of-pocket costs and US$4.7 in productivity losses due to the need for eight days of caregiving. Mean health system costs were US$2.6 for an outpatient visit and US$44.0 for inpatient treatment. The cost differed significantly across health system levels ranging from US$1.0 in the community-based programme to US$11.0 for a regional hospital outpatient visit, and from US$42.8-50.8 for inpatient stays.

**Conclusion:**

Malaria management imposes a substantial economic burden on households and the health system, particularly when requiring hospital admission. The household costs for a single malaria episode were equivalent to 44% of an individual’s income for a month, while the health system costs per episode were nearly half the current government health expenditure per capita. Considering that some children will have multiple episodes per year, these are substantial costs. Further investment in preventive strategies and the introduction of new interventions, such as vaccines, could release government health resources for other diseases while enabling economic growth for households.

**Supplementary Information:**

The online version contains supplementary material available at 10.1186/s12936-025-05684-0.

## Background

Despite being a preventable and treatable illness, malaria remains a major public health problem with 3.3 billion people at risk of infection globally [1]. In 2023, malaria cases reached 263 million with an estimated 597,000 deaths [2], with 94% of these cases occurring in Africa [2, 3]. Within a population of 12.9 million in 2022, Burundi recorded 8.2 million confirmed malaria cases, diagnosed using either rapid diagnostic tests or microscopy, representing an increase of 19% from 2021 [4]. The annual incidence rate of malaria remains high with 540 per 1000 inhabitants, and a case fatality rate of 0.018% in health centres and 0.74% in hospitals in 2022 [4] Malaria was responsible of 59% of health facility outpatient visits across all age groups in 2018 and is the largest cause of mortality for children under the age of five years [5].

Early malaria diagnosis and effective treatment is important to prevent deaths [3]. Burundi case management guidelines follow World Health Organization (WHO) recommendations, requiring diagnosis via a rapid diagnostic test (RDT) or microscopy before treatment with oral artemether lumefantrine for uncomplicated malaria and injectable artesunate for severe malaria [6, 7]. The Burundi government offers free malaria diagnosis and treatment at public health facilities to improve access to care [8]; however, patients still encounter barriers, such as sub-optimal case management and inadequate access to malaria prevention and treatment [9]. These barriers may include delayed or incorrect diagnosis, inconsistent adherence to treatment guidelines, and shortages of essential medicines or trained personnel, particularly in rural areas.

Burundi’s public healthcare system operates on a four-level structure: the community level, operational level (which includes health centres and district hospitals), intermediate level (comprising four regional hospitals), and central level (which includes four national hospitals) [6]. Alongside the national hospitals, private hospitals and clinics also contribute to healthcare services. Health centres serve as the entry point to the healthcare system and provide basic services. District hospitals act as the first referral point, offering more advanced curative treatments. Regional hospitals serve as the second referral level, providing specialized care, while the National Referral Hospital delivers highly specialized services.

While most of the healthcare has historically been based at health centres and hospitals, the Burundi government established a community-based management programme called integrated community case management (iCCM) in 2013 to improve timely access to treatment for hard-to-reach patients and reduce costs for children under five [10]. This community-level programme comprises trained volunteer community health workers (CHWs) who serve as the first point of contact for basic healthcare services, especially in remote or underserved areas. CHWs conduct health promotion, disease prevention, and basic curative care activities directly at the household level.

The community-based management initially focused on uncomplicated malaria, with CHWs trained to use rapid diagnostic tests (RDTs) to diagnose malaria and treat positive cases with artemisinin-based combination therapy (ACT). In subsequent years, the iCCM package was expanded to include pneumonia and diarrhoea, equipping CHWs to assess respiratory rates and administer amoxicillin for pneumonia and provide oral rehydration salts (ORS) and zinc for diarrhoea. CHWs are also trained to recognize danger signs requiring referral, and to deliver preventive health education in the community. The programme was expanded in 2021 to offer care for all age groups; however, it has not been fully implemented as only nine out of 42 districts service all age groups [6]. The scale-up of the programme has been hindered by limited funding, which is largely reliant on international organizations [6]. Despite the programme’s goals of lowering costs, a comprehensive large-scale study comparing the costs of community-based care versus health facility-based care has not been previously conducted.

While the clinical burden of malaria in Burundi is well understood [4, 8, 11], the economic burden remains relatively unquantified. One recent study estimated the cost per of malaria case in children under the age of five years at one national hospital for outpatient visits and inpatient treatment in the economic capital city (Bujumbura) to be $23.5 and $218.2, respectively, in 2019 international dollars [12]. The single setting limits the generalizability to the rest of Burundi.

Alongside nine other African countries, Burundi was selected for the second phase of vaccine rollout of RTS,S after the pilot programme due to its high malaria transmission [13]. As part of the pre-implementation evaluation process for the vaccine rollout, this study was conducted to estimate the economic burden of malaria among children under the age of five from a societal perspective, capturing both household costs and health system costs in Burundi.

## Methods

### Overview of study design

This study was designed as a prospective cost-of-illness using a micro costing approach. Cost data collected cover the entire episode of malaria from symptom onset until complete recovery or death from the illness episode, from both household and health system perspectives. Study sites were purposively selected to include at least one community implementing the iCCM programme, one health centre, and one hospital from each of the country’s five geographical regions: Eastern, Western, Central, Southern, and Northern. This led to a total of 15 sites comprising of five iCCM communities, five health centres, four district hospitals, and one regional hospital. Of these facilities, 11 were government-operated, two were privately owned, and two were faith-based.

### Study population and criteria

All patients under the age of five years who attended the study sites from January to February 2024 with malaria confirmed by either microscopy or RDT were eligible for inclusion. Costs associated with diagnostic tests that returned negative results were not included in the analysis. Informed consent was given by caregivers during study enrolment. Patients whose medical records were not found, or whose caregivers were unable to communicate with the investigator in Kirundi, French, or English were excluded.

### Cost data collection

Household costs were collected through interviews with patient caregivers using a structured cost-of-illness questionnaire [14]. Caregivers were interviewed three times for inpatients and twice for outpatients. For all outpatient locations (including iCCM), patient’s caregivers were asked about all direct and indirect costs incurred by the household before, during and after outpatient visit or admission. Direct medical costs included out-of-pocket expenditures for the cost of medicines, diagnostic and investigative tests, and medical services. Direct non-medical costs included transportation and meals while treatment-seeking. Indirect costs included productivity losses due to caregiving.

Health system costs were determined through the review of medical and financial records. Data were extracted from each patient’s electronic medical records at hospitals and paper-based medical records at health centres. Data extracted from medical records included the dates of outpatient visit or inpatient stay, clinical data on outcomes, and quantities of consumables used. The cost of services provided by CHWs (including the cost of their time, training, transportation to report and procure required medical supplies) in the iCCM programme was derived from a previous report evaluating the programme in Burundi [10]. The cost of consumables, tests, and medications for iCCM was assumed to be the same as at health centres, as these items are procured from them.

Ethical approval was granted by the Burundi National Ethics Committee (CNE/22/2023) and Human Research Ethics Committee of the University of Melbourne, Reference Number: 2023-27264-45509-5.

### Cost valuation

Costs were collected in local currency and then converted to 2023 United States dollars (US$) using an exchange rate of 2574 Burundian Francs (BIF) per US$ [15]. Unit costs for consumables were obtained from the financial department in health facility. Total costs were computed by multiplying the unit cost by the quantity of resources used.

The human capital approach was used to measure and value household productivity losses due to caregiving [16]. In the base case analysis, self-reported revenue losses were used. For individuals who did not indicate productivity-related revenue losses, productivity was assumed to be zero. A sensitivity analysis to estimate household productivity losses associated with caregiving time was further conducted. Since nationally representative daily income data for Burundi were unavailable, gross national income (GNI) per capita per day was used as a proxy, based on World Bank estimates for the study year [15]. The daily rate was calculated by dividing the annual per capita value by 365 days. It was assumed that all reported missed days represented full working days and that the economic value of time lost was equivalent across individuals, regardless of occupation. The total household productivity losses were then estimated by multiplying the daily rate by the number of working days missed by both caregivers and other family members involved in supporting the primary caregivers.

Unit costs for outpatient visits and bed days were valued using the World Health Organization Choosing Interventions That Are Cost-Effective (WHO-CHOICE) estimates [17]. These WHO‑CHOICE unit costs for outpatient visits and inpatient bed‑days capture the facility‑level overhead and general service delivery cost components [18]. For inpatient bed‑days, this includes personnel, capital infrastructure and equipment, laboratory, maintenance and other operational costs of the hospital, as well as food costs, but excludes disease‑specific inputs such as drugs and diagnostic tests. Similarly, outpatient unit costs include personnel, capital infrastructure and equipment, laboratory, maintenance and other operational costs of the facility, but exclude disease‑specific costs. These estimates were inflated to 2023 values using the consumer price index for Burundi [19].

The total health system cost was calculated as the sum of the WHO‑CHOICE–based cost of health service delivery (outpatient visit cost or inpatient bed‑day cost) and the direct medical costs extracted from patient records, which included medicines, diagnostic tests, consumables, and other disease‑specific medical procedures. For the iCCM programme, the cost of CHW service delivery was obtained separately from a local pilot implementation evaluation. The total health system cost for iCCM was then calculated as the sum of the CHWs service delivery cost and the cost of medical consumables, rapid diagnostic tests (RDTs), and medications used.

The societal cost was computed as the sum of total cost household cost and health system cost. To estimate the nationwide mean societal cost of a malaria episode in Burundi, national‑level health‑seeking behaviour patterns from the country’s Annual Health Statistics Report were used [4]. This report provides the distribution of malaria cases managed at each level of the health system, including community‑level care (iCCM), health centres, and hospitals. Because the report does not distinguish between hospital types (district vs. regional), the distribution of hospital attendance observed within the main study, conducted during the same period, was used to estimate the cost per episode managed at hospitals. The national‑level health‑seeking behaviours data to the corresponding cost estimates for each level of care was applied to calculate the weighted average nationwide societal cost per malaria episode.

### Data analysis

All data were collated in REDCap, and Stata version 18 was used for data cleaning and analysis. Continuous variables including age, length of stay, episode day, duration of the episode before consultation, and cost were presented using mean, standard deviations (SD), median, and inter-quartile ranges. Discrete variables including gender, type of visit, diagnosis, discharge outcome, health resources and services use were presented using frequency and proportions. As the cost data were skewed, Mann–Whitney U tests were used when comparing two health facility levels and Kruskal Wallis tests when comparing more than two health facility levels [20]. Chi-square tests were used to compare discrete variables across health facility levels. A p-value of 0.05 was used as the threshold for statistical significance [21].

To address missing data, mean imputation was applied using the average values from participants with the same type of visit at the same study site. Sensitivity analyses were conducted using complete case analysis and multiple imputation by chained equations to assess the robustness of the estimates [22].

To assess the factors influencing costs, a generalized linear model (GLM) with a gamma link function was applied to cases with complete data. Potential independent variables based on the availability of data and literature [12, 23, 24], including gender, age, type of visits, delay in seeking healthcare, and the employment and education levels of caregivers were explored. Exponentiated coefficients, interpreted as the ratios of the arithmetic means between the categories of the explanatory variables, holding all other variables constant, were also estimated. Further details about model assumptions and choice of potential variables are provided in supplementary materials. 

## Results

### Participants and household characteristics

A total of 622 participants were enrolled in the study with an average age of 25 months (SD = 15). This included 218 (35%) inpatient stays and 404 (65%) outpatient visits. Overall, a quarter of patients (153/622) were managed in the iCCM programme and the remaining participants were managed at health facilities (Table 1). Participants sought healthcare three days after symptom onset (SD = 15), and their malaria episodes lasted nine days (SD = 15) from the onset of symptoms to recovery. The average length of inpatient stay was six days (SD = 3). Two participants that were hospitalized died from their malaria infection. The majority of caregivers were the patient’s mother (93%, 580/622), had no or primary level education (85%, 531/622), and were farmers (93%, 579/622; Table 2).

**Table 1:** Demographic and clinical characteristics of patients enrolled in the study

Characteristic	iCCM (n = 153)	Health center (n = 220)	District hospital (n = 211)	Regional hospital (n = 38)	Total (N = 622)
Age in months, mean (SD)	28 (15)	26 (16)	20 (14)	25 (14)	25 (15)
Female, n (%)	89 (58%)	117 (53%)	102 (48%)	13 (34%)	321 (52%)
Days before seeking treatment, mean (SD)	1 (1)	2 (2)	5 (24)	2 (2)	3 (15)
Episode duration in days, mean (SD)	5 (1)	7 (3)	13 (25)	13 (4)	9 (15)
Admitted for inpatient care, n (%)	–	–	186 (88%)	32 (84%)	218 (35%)
Length of stay for inpatient care in days, mean (SD)	–	–	6 (3)	8 (3)	6 (3)
Discharge outcome for inpatient stays n (%)					
Recovered	–	–	32 (17%)	1 (3%)	33 (16%)
Partially recovered	–	–	139 (75%)	31 (97%)	170 (83%)
Transfer to another facility	–	–	1 (1%)	0 (0%)	1 (0%)
Died	–	–	2 (1%)	0 (0%)	2 (1%)
Not reported			12 (6%)	0 (0%)	16 (3%)

*iCCM* integrated community case management, *SD* standard deviation

**Table 2:** Demographic characteristics of the patients’ caregivers

Characteristic	iCCM (n = 153)	Health center (n = 220)	District hospital (n = 211)	Regional hospital (n = 38)	Total (N = 622)
Relationship with patient (N = 622), n (%)					
Mother	139 (91%)	203 (92%)	202 (96%)	36 (95%)	580 (93%)
Father	8 (5%)	12 (5%)	3 (1%)	0 (0%)	23 (4%)
Grandparent	1 (1%)	1 (0%)	5 (2%)	2 (5%)	9 (1%)
Sibling	2 (1%)	4 (2%)	1 (1%)	0 (0%)	7 (1%)
Aunt/uncle	2 (1%)	0 (0%)	0 (0%)	0 (0%)	2 (0%)
Cousin	1 (1%)	0 (0%)	0 (0%)	0 (0%)	1 (0%)
Patients’ mother education level (N = 580), n (%)					
None	104 (68%)	108 (49%)	79 (37%)	5 (13%)	296 (48%)
Primary school	42 (27%)	83 (38%)	95 (45%)	15 (39%)	235 (38%)
Secondary school or equivalent	6 (4%)	18 (8%)	24 (11%)	9 (24%)	57 (9%)
High school	1 (1%)	8 (4%)	13 (6%)	8 (21%)	30 (5%)
Bachelor’s degree and above	0 (0%)	1 (0%)	0 (0%)	1 (3%)	2 (0%)
Others	0 (0%)	2 (1%)	0 (0%)	0 (0%)	2 (0%)
Patients’ mother occupation (N = 580), n (%)					
Farmer/agriculture	149 (97%)	208 (95%)	192 (91%)	30 (79%)	579 (93%)
Public employee	2 (1%)	6 (3%)	3 (1%)	3 (8%)	14 (2%)
Private employee	0 (0%)	2 (1%)	1 (0%)	0 (0%)	3 (0%)
Unemployed	1 (1%)	3 (1%)	4 (2%)	2 (5%)	10 (2%)
Others	1 (1%)	1 (0%)	11 (5%)	3 (8%)	16 (2%)
Household time loss in days, mean (SD)	3 (3)	3 (3)	13 (9)	18 (12)	7 (9)

*iCCM* integrated community case management, *n/a* not applicable, *SD* standard deviation

### Household costs

Overall, the mean cost per outpatient visit for malaria to the household was US$7.3 and US$45.1 for inpatient stays. The household costs varied substantially across healthcare levels, ranging from US$5.3 at iCCM to US$20.6 at the regional hospital (Table 3). For inpatient stays, the costs were $41.8 for district hospital as compared to US$63.9 for regional hospital.

**Table 3:** Mean cost and standard deviation per outpatient and inpatient malaria episode by type of health facility and cost component in 2023 United States dollars

Cost component	Outpatient					Inpatient		
	iCCM (n = 153)	Health center (n = 220)	District hospital (n = 25)	Regional hospital (n = 6)	Overall (n = 404)	District hospital (n = 186)	Regional hospital (n = 32)	Overall (n = 218)
Total household cost	**5.3 (5.5)**	**6.7 (5.6)**	**22.0 (16.2)**	**20.6 (21.0)**	**7.3 (8.2)**	**41.8 (30.3)**	**63.9 (51.4)**	**45.1 (35.0)**
Direct medical expenses	0.0 (0.1)	0.2 (0.6)	2.2 (2.9)	2.6 (2.8)	0.3 (1.1)	2.6 (4.1)	8.5 (12.5)	3.5 (6.4)
Medications	0.0 (0.1)	0.1 (0.6)	1.7 (2.5)	2.6 (2.8)	0.2 (1.0)	2.5 (4.0)	8.3 (12.5)	3.4 (6.3)
Investigation	0.0 (0.0)	0.0 (0.2)	0.5 (1.7)	0.0 (0.0)	0.0 (0.5)	0.1 (0.5)	0.0 (0.0)	0.1 (0.5)
Consultation/services cost	0.0 (0.0)	0.0 (0.0)	0.0 (0.0)	0.0 (0.0)	0.0 (0.0)	0.0 (0.3)	0.2 (0.8)	0.1 (0.4)
Direct non-medical expenses	1.1 (2.1)	3.0 (2.9)	9.0 (6.5)	5.9 (2.0)	2.7 (3.5)	14.5 (10.4)	19.9 (15.1)	15.3 (11.4)
Transportation	0.3 (1.1)	0.5 (1.2)	4.3 (5.3)	2.3 (1.0)	0.7 (2.0)	4.9 (4.6)	4.4 (4.8)	4.8 (4.6)
Meal	0.7 (1.7)	2.4 (2.2)	4.6 (2.0)	3.4 (1.8)	1.9 (2.3)	8.5 (7.5)	14.3 (10.6)	9.4 (8.3)
Accommodation	0.0 (0.2)	0.1 (0.6)	0.0 (0.1)	0.0 (0.1)	0.1 (0.5)	0.1 (0.6)	0.0 (0.2)	0.1 (0.6)
Other episode related expenses	0.1 (0.4)	0.1 (0.4)	0.1 (0.4)	0.2 (0.5)	0.1 (0.4)	0.9 (2.1)	1.2 (2.7)	0.9 (2.2)
Productivity losses	4.1 (4.7)	3.5 (3.8)	10.8 (9.6)	12.0 (21.8)	4.3 (5.6)	24.7 (22.2)	35.4 (30.4)	26.3 (23.8)
Total health system cost	**1.0 (0.1)**	**2.8 (2.5)**	**8.4 (7.7)**	**11.0 (2.7)**	**2.6 (3.4)**	**42.8 (18.2)**	**50.8 (23.3)**	**44.0 (19.2)**
Drugs and medical supplies	0.6 (0.1)	2.1 (2.5)	6.2 (7.1)	7.6 (3.6)	1.9 (3.0)	33.5 (16.5)	34.7 (19.1)	33.7 (16.8)
Investigations	0.3 (0.0)	0.3 (0.2)	1.6 (1.0)	2.9 (2.6)	0.4 (0.6)	2.2 (2.3)	6.9 (5.5)	2.9 (3.4)
Routine services (visit/bed days)	0.1 (0.0)	0.4 (0.0)	0.5 (0.0)	0.5 (0.0)	0.3 (0.1)	7.1 (3.6)	9.3 (3.1)	7.4 (3.6)
Total societal cost	**6.3 (5.5)**	**9.5 (6.2)**	**30.4 (22.4)**	**31.7 (20.0)**	**9.9 (10.3)**	**84.6 (36.2)**	**114.7 (65.7)**	**89.1 (43.0)**

*iCCM* integrated community case management

Productivity losses were the largest household cost component, accounting for 59% (US$4.3) of total household costs for outpatient visits and 58% (US$26.3) of costs for inpatient stays. Direct non-medical expenses were the next major driver, accounting for 37% (US$2.7) of outpatient costs and 34% (US$15.3) of inpatient costs. Direct medical expenses accounted for a smaller share, comprising 4% (US$0.3) of outpatient costs and 8% (US$3.5) of inpatient costs. See Table S2 and S3 for corresponding medians and interquartile ranges.

### Health system costs

Overall, the mean health system cost per malaria episode was US$2.6 for outpatients and US$44.0 for inpatients (Table 3). These costs differed greatly by healthcare level, ranging from US$1.0 at iCCM to US$11.0 at regional hospital for outpatient and from US$42.8 at district hospital to US$50.8 at regional hospital (Table 4). Medications accounted for most of the health system costs, amounting to US$1.9 (73%) for outpatient and US$33.7 (77%) for inpatient, followed by the visit costs of US$0.3 (12%) for outpatient and US$7.4 (17%) for inpatient.

**Table 4:** Mean and median cost per episode by health facility and visit type in 2023 United States dollars

Visit type	iCCM (n = 153)	Health center (n = 220)	District hospital (n = 211)	Regional hospital (n = 38)	Overall (N = 622)	p-value
Outpatient						
Mean (SD)	6.3 (5.5)	9.5 (6.2)	30.4 (22.4)	31.7 (20.0)	9.9 (10.3)	< 0.001^a^
Median (IQR)	4.9 (2.7–7.7)	8.1 (5.1–11.5)	25.2 (19.0–33.6)	26.4 (20.1–29.1)	7 (4.2–11.8)	
Inpatient						
Mean (SD)	–	–	84.6 (36.2)	114.7 (65.7)	89.1 (43.0)	0.022^b^
Median (IQR)	–	–	81.0 (59.2–102.1)	92.3 (74.1–132.2)	82 (60.7–104.6)	
Overall^c^						
Mean (SD)	6.3 (5.5)	9.5 (6.2)	78.2 (39.0)	101.6 (67.9)	37.7 (46.3)	< 0.001^a^
Median (IQR)	4.9 (2.7–7.7)	8.1 (5.1–11.5)	74.7 (51.9–97.0)	83.7 (58.1–115.3)	12.5 (5.6–64.5)	

*iCCM* integrated community case management, *IQR* interquartile range, *SD* standard deviation

^a^A Kruskal Wallis test was used to compare between iCCM, health center, district hospital and regional hospital

^b^A Mann–Whitney test was used to compare the difference between district and regional costs for inpatient cases

^c^indicates the cost per episode at health facilities level regardless of visit type

### Societal costs

**Fig. 1:**
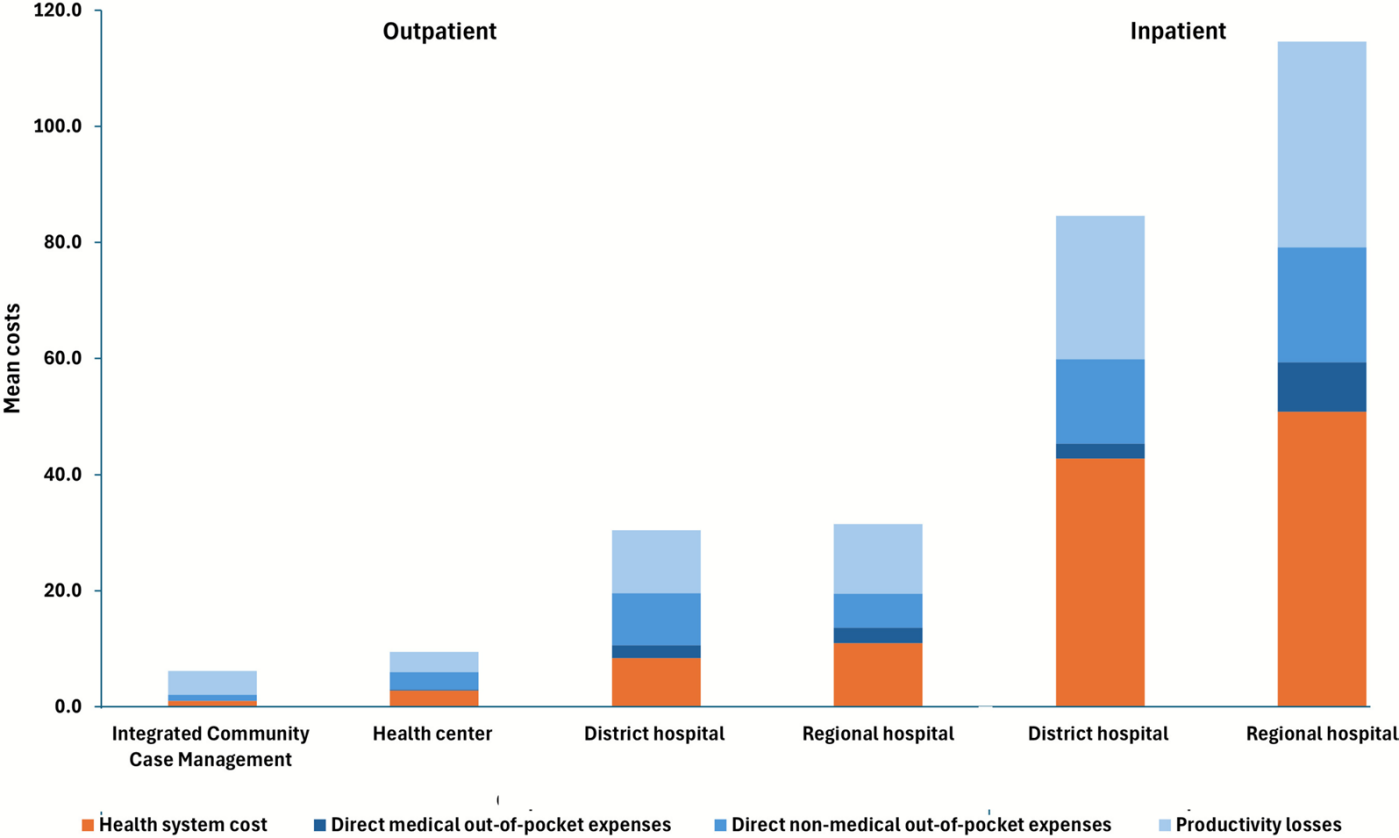
Distribution of societal cost by cost components in 2023 United States dollars

The mean societal cost per episode of malaria, obtained by combining the household and health system costs and weighting by nationwide health-seeking behaviour was US$12.1. The societal cost per outpatient amounted to US$ 6.3 at the community level, US$ 9.5 at health centres, US$ 30.4 at district hospitals, and US$ 31.7 at regional hospitals (Table 4). Outpatient costs at hospitals were significantly higher compared to iCCM and health centres; this was mostly driven by the high cost of transportation, additional meals required during healthcare seeking, and the high provider costs, which increased at each higher level of the healthcare system (Fig. 1). For inpatient stays, district hospitals cost US$84.6 while the regional hospital cost US$114.7.

### Factors influencing costs

When performing regression analysis using the GLM, the societal cost per episode significantly increased due to a higher level of healthcare facilities, a longer time elapsing before seeking healthcare, when utilizing inpatient hospitalization, and when the main caregiver’s employment was outside of agriculture (Table 5).

**Table 5:** Generalized linear model regression results for the predictors of the total societal cost per malaria episode in 2023 United States dollars (N = 526)

	Coefficient	Standard error	Ratio of arithmetic means	p-value	95% confidence interval
Age	− 0.001	0.002	0.999	0.545	− 0.005 to 0.003
Sex: female as reference	− 0.008	0.057	0.992	0.883	− 0.121 to 0.104
Healthcare level: iCCM as reference					
Primary care	0.294	0.074	1.342	< 0.001	0.149 to 0.438
Secondary care	1.453	0.136	4.276	< 0.001	1.185 to 1.720
Regional care	1.573	0.171	4.821	< 0.001	1.238 to 1.909
Patient type: outpatient as reference	1.020	0.128	2.773	< 0.001	0.768 to 1.271
Time to consult with waiting ≤ 2 days as reference	0.271	0.074	1.311	< 0.001	0.127 to 0.416
Education level of mother: No formal education	0.005	0.062	1.005	0.933	− 0.116 to 0.127
Profession of mother: Agriculturist/farmer as reference	0.361	0.117	1.435	0.002	0.133 to 0.590

*iCCM* integrated community case management

### Sensitivity analysis

The sensitivity analysis evaluating the impact of missing data revealed that excluding participants with incomplete data consistently resulted in similar estimates (Table S4) compared to the base case, which employed a mean imputation approach for handling missing values. Highest variations were observed with inpatient costs which increased by 2% at district hospital and decreased by 6% at regional hospital. Using multiple imputations produced slightly higher estimates as compared to the base case with the exceptions of lower outpatient costs at district hospitals, and the outpatient costs at regional hospitals, which remained the same (Table S5). The greatest variations were the health centre outpatient costs, which increased by 39%, and the district hospital outpatient costs, which decreased by 36%.

When caregiver time was valued using the GNI-based approach, productivity losses decreased in importance, representing 44% (US$2.4) of total household costs for outpatient visits and 37% (US$11.2) of costs for inpatient stays (Table S6). At the societal level, this approach resulted in lower total costs across all facility types, with reductions of approximately 12–30% compared to the base case. The largest difference was observed for regional hospital outpatient episodes, where societal costs were nearly 30% lower (US$22.2 vs. US$31.7 in the base case). Despite these reductions in magnitude, the overall cost ranking was unchanged: iCCM episodes had the lowest societal costs, while inpatient care at regional hospitals had the highest.

## Discussion

As part of the pre-implementation evaluation process for the second phase of the RTS,S vaccine rollout, this study is the first to comprehensively assess the economic burden of malaria among Burundian children under five. The results from this study encompass all regions and levels of healthcare, providing robust economic evidence on cost per malaria episode in Burundi. The health system cost per episode of US$4.1 is nearly half the Burundi government healthcare budget allocation per capita in 2022–2023 (US$8.7) [25]. While the entire healthcare budget for Burundi is larger than this due to co-financing from development partners, this figure indicates that substantial resources are needed for malaria case management for the eight million malaria cases annually [4].

Despite existing policies designed to offer free healthcare through the formal health system, households in Burundi continue to bear substantial direct and indirect costs, particularly hospitalized malaria episodes. On average, the household cost per episode was US$8.0, consisting of US$3.3 in direct costs and US$4.7 in productivity losses. These mean overall costs are equivalent to 44% of the monthly per capita income (US$18.0) in Burundi [26]. These findings are higher than previous findings from neighbouring Democratic Republic of Congo (DRC), where the mean household cost per uncomplicated malaria episode in children under 14 years was about seven times higher than the daily minimum wage [27].

A substantial part of household costs (59%) were the indirect costs borne by economically active household members, who had to pause regular activities to take care of their sick child and access medical services. On average, participant’s carers missed work for eight days throughout the duration of a malaria episode. As reported in other African countries, malaria often leads to productivity losses for the entire family due to absenteeism from daily activities [23]. Malaria episodes have been associated with average losses of 7 days in DRC [27] and 9 days in Malawi [23]. In highly endemic countries like Burundi, where malaria recurrence in children can reach four episodes per year [28], this would mean up to 32 days lost per child, hindering economic development and impoverishing families. Poverty can create and sustain conditions that perpetuate malaria transmission within the population, perpetuating a vicious cycle between malaria and economic hardship [29].

The mean societal cost per episode of malaria was US$12.1, which is equivalent to 6% of the 2023 Burundian GDP per capita (US$200), demonstrating the substantial economic impact of malaria on economic development and growth. Examining costs by visit type, the mean societal cost per outpatient visit was US$9.9, while the cost per inpatient visit was US$89.1. These findings align closely with previous societal cost estimates from the western region (Bujumbura) in Burundi of US$9.7 per outpatient visit and US$90.4 per inpatient visit (values converted and inflated to 2023 US$) [12].

The societal cost per episode significantly increased with higher levels of healthcare facilities. Costs were significantly lower if malaria was managed in communities compared to health facilities. Managing malaria at health facilities resulted in 34–382% higher costs than at the iCCM. These results could be attributed to higher expenses at higher-level facilities, which are often located in urban areas farther from households, where the cost of living and basic goods tends to be higher. These findings highlight the importance of the iCCM programme in reducing the cost of illness for households while alleviating health system shortfalls of qualified healthcare professionals [9].

While results from this study demonstrate the cost-saving potential of community-based malaria care compared to health facilities, it is important to interpret these findings in context. From a health system perspective, the services delivered at different levels of care are not directly comparable. CHWs under the iCCM programme are mandated to treat only uncomplicated malaria cases, while health centres, outpatient departments, and hospitals manage increasingly complicated or severe cases requiring more advanced diagnostics and treatments. As such, the higher unit costs observed at facility levels partly reflect the greater clinical complexity and resource intensity of care provided. From a policy perspective, expanding community‑based case management for uncomplicated malaria can improve access, reduce treatment costs, and lower households’ non‑medical expenses such as transportation and meals during care‑seeking, while reducing pressure on higher‑level facilities. At the same time, timely referral of suspected severe or complicated cases to appropriate facility‑level care remains critical to ensuring optimal outcomes.

Addressing delays before seeking healthcare is essential for reducing the cost of malaria. In this study, the total cost increased with longer delays in seeking healthcare. This observation is consistent with findings from other endemic regions where delayed diagnosis in uncomplicated malaria contributes to extended recovery periods and increased costs [8, 27, 30]. Results from the GLM indicate that patients who sought care within two days of symptom onset had lower total costs by 31% compared to patients who sought care after two days. Hence, strengthening CHWs initiatives and improving communication and educational outreach could lower costs by encouraging prompt treatment during malaria episodes, and increase the likelihood of effective treatment, reducing the duration of illness, and leading to better health outcomes. Ultimately, preventing malaria remains the most effective way to minimize these costs.

This study has several limitations. First, due to limited resources to collect required population data, the study did not capture all age groups, though malaria affects everyone in Burundi. Second, this study did not include the costs of diagnostic tests that returned negative results. As a result, the total economic burden of malaria care may be underestimated, particularly from the health system perspective, since testing both positive and negative cases represents a routine and necessary part of malaria case management. Future studies could include the costs of all tests, regardless of result, in order to provide a more comprehensive estimate of the resources required for malaria diagnosis and surveillance. Third, this study was not able to collect unit costs for outpatient visits and bed days, instead relying on dated WHO-CHOICE estimates [17]. The quality of care and human resources might have changed since original publication, which likely results in underestimation of the actual cost of visit and hospital bed days. Fourth, as is common in prospective studies, there were instances of missing data that could potentially influence the accuracy of our estimates. To address this, multiple methods to handle missingness and conducted sensitivity analyses to assess the impact were employed. Importantly, these approaches yielded consistent and robust results, with minimal variation between them, suggesting that the missing data had only a slight effect on our findings.

## Conclusion

Malaria has a large health burden to households and the health system in Burundi. Each malaria episode diverted substantial household resources towards treatment while resulting in considerable time away from economic activity. This substantial burden underscores the urgent need for a more sustainable approach that prioritizes prevention over management. Understanding the unit costs for malaria in Burundi is an important first step in evaluating the costs and cost-effectiveness of new interventions, including the newly available RTS,S and R21 vaccines.

## Supplementary Information

Supplementary material 1.

## Data Availability

Data is provided within the manuscript or supplementary information files.
