# Supplementary material for: Economic burden of malaria in children under the age of five in Burundi
Source: Malar J. 2025 Dec 1;25:11. doi: 10.1186/s12936-025-05684-0 (PMC12777481; doi:10.1186/s12936-025-05684-0)
Supplement: Supplementary file 1 — Supplementary material 1. [file 12936_2025_5684_MOESM1_ESM.docx]

**SUPPLEMENTAL MATERIALS**

**Supplementary Text 1. Regression model**

To assess the factors influencing costs, a generalized linear model (GLM) with a gamma link function was applied to cases with complete data. The model explored all potential independent variables based on the availability of data and literature [1-3] including gender, age, type of visits, delay in seeking healthcare, and the employment and education levels of caregivers. Since this study did not collect data on disease severity, we used inpatient hospitalization as a proxy for severe malaria to assess its impact on cost. This approach assumed that uncomplicated malaria would typically be managed through outpatient visits, consistent with local guidelines [4] For delays in seeking healthcare, we classified two categories: less than 2 days and 2 days or more, as suggested by previous studies [3, 5] Given mothers were typically primary caregivers, our regression analysis focused exclusively on them to evaluate household factors influencing the cost. To enhance model clarity and focus, we then ran a simplified model using backward stepwise regression retaining a set of variables with p-values less than 0.05 (Table S1). In the GLM model with a gamma log-link, each coefficient (β) indicates the change in the logarithm of the expected mean cost associated with a one-unit increase in the respective independent variable, while keeping all other variables constant. For ease of interpretation, we further estimated and report exponentiated coefficients ( $e^{\beta}$) which give the multiplicative factor by which the cost changes.

**References**

1. Hennessee I, Chinkhumba J, Briggs-Hagen M, Bauleni A, Shah MP, Chalira A, et al. Household costs among patients hospitalized with malaria: evidence from a national survey in Malawi, 2012. Malaria Journal. 2017;16(1):395.

2. Tefera DR, Sinkie SO, Daka DW. Economic Burden of Malaria and Associated Factors Among Rural Households in Chewaka District, Western Ethiopia. Clinicoecon Outcomes Res. 2020;12:141-52.

3. Hezagira N, Youngkong S, Riewpaiboon A. Economic burden of malaria in Burundian children: An evidence for disease management in resource limited settings. Life Sciences, Medicine and Biomedicine. 2022;6.

4. USAID. Burundi malaria profile [cited 2024 September 13 ]. Available from: <https://www.pmi.gov/wp-content/uploads/2024/04/FY-2024-Burundi-Country-Malaria-Profile.pdf>.

5. Trampuz A, Jereb M, Muzlovic I, Prabhu RM. Clinical review: Severe malaria. Critical Care. 2003;7(4):315.

**Supplementary Table 1. Generalised linear model regression results for the full list of societal cost predictors using final model (N=526).**

| **Predictors** | **Coefficient** | **Standard error** | **Ratio of arithmetic  means (exponentiated coefficient)** | **p-value** | **95% Confidence interval** |
| --- | --- | --- | --- | --- | --- |
| Healthcare level: iCCM as reference |  |  |  |  |  |
| Primary care | 0.293 | 0.072 | 1.340 | <0.001 | 0.152 to 0.434 |
| Secondary care | 1.46 | 0.13 | 4.306 | <0.001 | 1.205 to 1.715 |
| Regional care | 1.575 | 0.163 | 4.831 | <0.001 | 1.256 to 1.894 |
| Patient type: outpatient as reference | 1.019 | 0.127 | 2.770 | <0.001 | 0.771 to 1.267 |
| Time to consult with waiting ≤2 days as reference | 0.272 | 0.072 | 1.313 | <0.001 | 0.131 to 0.414 |
| Profession of mother: Agriculturist/farmer as reference | 0.366 | 0.115 | 1.442 | 0.001 | 0.141 to 0.591 |

**Supplementary Table 2. Median cost per outpatient malaria episode by health facility type and cost component with interquartile range in 2023 United States dollars.**

| **Cost component** | **iCCM (n=153)** | **Health center (n=220)** | **District hospital (n=25)** | **Regional hospital (n=6)** | **Overall (N=404)** |
| --- | --- | --- | --- | --- | --- |
| **Total household cost** | **3.9 (1.7 - 6.8)** | **5.2 (3.0 - 8.4)** | **19.3 (11.1 - 26.6)** | **13.2 (10.2 - 18.1)** | **5.1 (2.7 - 8.9)** |
| Direct medical out-of-pocket expenses | 0.0 (0.0 - 0.0) | 0.0 (0.0 - 0.0) | 1.2 (0.0 - 2.5) | 2.2 (0.0 - 4.5) | 0.0 (0.0 - 0.0) |
| Medications | 0.0 (0.0 - 0.0) | 0.0 (0.0 - 0.0) | 1.2 (0.0 - 2.5) | 2.2 (0.0 - 4.5) | 0.0 (0.0 - 0.0) |
| Investigation | 0.0 (0.0 - 0.0) | 0.0 (0.0 - 0.0) | 1.2 (0.0 - 1.9) | 2.2 (0.0 - 4.5) | 0.0 (0.0 - 0.0) |
| Consultation/services cost | 0.0 (0.0 - 0.0) | 0.0 (0.0 - 0.0) | 0.0 (0.0 - 0.0) | 0.0 (0.0 - 0.0) | 0.0 (0.0 - 0.0) |
| Direct non-medical out-of-pocket expenses | 0.0 (0.0 - 0.0) | 0.0 (0.0 - 0.0) | 0.0 (0.0 - 0.0) | 0.0 (0.0 - 0.0) | 0.0 (0.0 - 0.0) |
| Transportation | 0.6 (0.0 - 1.6) | 2.3 (1.2 - 4.2) | 7.0 (5.8 - 9.3) | 6.6 (4.3 - 7.4) | 1.6 (0.5 - 3.5) |
| Meal | 0.0 (0.0 - 0.0) | 0.0 (0.0 - 0.4) | 2.7 (1.2 - 4.5) | 2.0 (1.6 - 3.1) | 0.0 (0.0 - 0.6) |
| Accommodation | 0.0 (0.0 - 1.0) | 1.9 (0.9 - 3.2) | 4.9 (3.3 - 6.2) | 3.6 (2.3 - 4.9) | 1.2 (0.0 - 2.7) |
| Other episode related expenses | 0.0 (0.0 - 0.0) | 0.0 (0.0 - 0.0) | 0.0 (0.0 - 0.0) | 0.0 (0.0 - 0.0) | 0.0 (0.0 - 0.0) |
| Productivity losses | 2.9 (0.8 - 5.8) | 2.3 (1.6 - 4.0) | 8.5 (4.9 - 15.0) | 2.9 (1.2 - 9.3) | 2.9 (1.5 - 5.4) |
| **Total health system cost** | **1.0 (1.0 - 1.0)** | **1.7 (1.6 - 3.3)** | **6.3 (4.4 - 8.1)** | **10.6 (8.5 - 13.7)** | **1.6 (1.0 - 2.2)** |
| Drugs and medical supplies | 0.6 (0.6 - 0.6) | 1.0 (0.9 - 2.4) | 3.9 (3.5 - 5.7) | 6.9 (4.8 - 9.1) | 0.9 (0.6 - 1.4) |
| Investigation cost | 0.3 (0.3 - 0.3) | 0.3 (0.3 - 0.3) | 1.6 (0.9 - 2.2) | 2.5 (0.9 - 5.0) | 0.3 (0.3 - 0.3) |
| Routine services (visit/bed days) | 0.1 (0.1 - 0.1) | 0.4 (0.4 - 0.4) | 0.5 (0.5 - 0.5) | 0.5 (0.5 - 0.5) | 0.4 (0.1 - 0.4) |
| **Total societal cost** | **4.9 (2.7-7.7)** | **8.1 (5.1-11.5)** | **25.2 (19.0-33.6)** | **26.4 (20.1-29.1)** | **7 (4.2-11.8)** |

iCCM: integrated community case management

**Supplementary Table 3. Median cost per inpatient malaria episode by health facility type and cost component with interquartile range in 2023 United States dollars.**

| **Cost component** | **District hospital (n=186)** | **Regional hospital (n=32)** | **Overall (n=218)** |
| --- | --- | --- | --- |
| **Total household cost** | **33.7 (18.5 - 53.5)** | **46.8 (34.8 - 75.3)** | **35.4 (20.2 - 56.1)** |
| Direct medical out-of-pocket expenses | 1.0 (0.0 - 3.3) | 4.3 (0.5 - 12.6) | 1.0 (0.0 - 4.1) |
| Medications | 0.8 (0.0 - 3.2) | 4.2 (0.5 - 12.6) | 1.0 (0.0 - 3.9) |
| Investigation | 0.0 (0.0 - 0.0) | 0.0 (0.0 - 0.0) | 0.0 (0.0 - 0.0) |
| Consultation/services cost | 0.0 (0.0 - 0.0) | 0.0 (0.0 - 0.0) | 0.0 (0.0 - 0.0) |
| Direct non-medical out-of-pocket expenses | 10.5 (7.6 - 19.6) | 17.2 (9.1 - 24.4) | 11.8 (7.7 - 20.4) |
| Transportation | 3.9 (1.9 - 6.2) | 3.1 (1.4 - 5.5) | 3.7 (1.9 - 6.2) |
| Meal | 6.5 (3.9 - 10.3) | 11.6 (7.4 - 19.4) | 6.9 (4.3 - 11.1) |
| Accommodation | 0.0 (0.0 - 0.0) | 0.0 (0.0 - 0.0) | 0.0 (0.0 - 0.0) |
| Other episode related expenses | 0.0 (0.0 - 0.8) | 0.0 (0.0 - 0.6) | 0.0 (0.0 - 0.8) |
| Productivity losses | 16.3 (8.7 - 33.0) | 26.0 (16.0 - 39.6) | 18.0 (9.7 - 35.0) |
| **Total health system cost** | **40.1 (32.4 - 51.6)** | **43.1 (33.2 - 59.5)** | **40.5 (32.4 - 53.0)** |
| Drugs and medical supplies | 31.7 (24.0 - 40.5) | 30.4 (20.5 - 43.2) | 31.3 (23.6 - 40.7) |
| Investigation cost | 1.0 (0.3 - 4.1) | 5.7 (4.1 - 7.1) | 1.6 (0.3 - 4.7) |
| Routine services (visit/bed days) | 5.9 (4.7 - 8.3) | 8.6 (7.4 - 9.9) | 7.1 (4.7 - 8.6) |
| **Total societal cost** | **81 (59.2-102.1)** | **92.3 (74.1-132.2)** | **82 (60.7-104.6)** |

**Supplementary Table 4. Mean cost and standard deviation per outpatient and inpatient malaria episode by type of health facility and cost component from sensitivity analysis using cases with complete data (2023 United States dollars).**

| **Cost component** | **Outpatient** | | | | | **Inpatient** | | |
| --- | --- | --- | --- | --- | --- | --- | --- | --- |
|  | **iCCM (n=153)** | **Health center (n=189)** | **District hospital (n=25)** | **Regional hospital (n=6)** | **Overall (n=373)** | **District hospital (n=159)** | **Regional hospital (n=30)** | **Overall (n=189)** |
| **Total household cost** | **5.3 (5.5)** | **6.7 (5.4)** | **22.0 (16.2)** | **20.6 (21.0)** | **7.4 (8.3)** | **42.1 (29.8)** | **61.1 (51.4)** | **45.1 (34.7)** |
| Direct medical out-of-pocket expenses | 0.0 (0.1) | 0.2 (0.7) | 2.2 (2.9) | 2.6 (2.8) | 0.3 (1.1) | 2.6 (4.1) | 8.7 (12.8) | 3.5 (6.7) |
| Medications | 0.0 (0.1) | 0.2 (0.7) | 1.7 (2.5) | 2.6 (2.8) | 0.2 (1.0) | 2.5 (4.0) | 8.5 (12.8) | 3.4 (6.6) |
| Investigation | 0.0 (0.0) | 0.0 (0.2) | 0.5 (1.7) | 0.0 (0.0) | 0.0 (0.5) | 0.1 (0.6) | 0.0 (0.0) | 0.1 (0.5) |
| Consultation/services cost | 0.0 (0.0) | 0.0 (0.0) | 0.0 (0.0) | 0.0 (0.0) | 0.0 (0.0) | 0.0 (0.1) | 0.2 (0.9) | 0.0 (0.4) |
| Direct non-medical out-of-pocket expenses | 1.1 (2.1) | 3.1 (3.0) | 9.0 (6.5) | 5.9 (2.0) | 2.7 (3.6) | 14.6 (10.7) | 19.1 (15.3) | 15.3 (11.6) |
| Transportation | 0.3 (1.1) | 0.5 (1.2) | 4.3 (5.3) | 2.3 (1.0) | 0.7 (2.0) | 4.9 (4.7) | 4.1 (4.8) | 4.8 (4.7) |
| Meal | 0.7 (1.7) | 2.5 (2.3) | 4.6 (2.0) | 3.4 (1.8) | 1.9 (2.3) | 8.8 (7.9) | 14.1 (10.8) | 9.6 (8.6) |
| Accommodation | 0.0 (0.2) | 0.1 (0.7) | 0.0 (0.1) | 0.0 (0.1) | 0.1 (0.5) | 0.1 (0.6) | 0.0 (0.2) | 0.1 (0.5) |
| Other episode related expenses | 0.1 (0.4) | 0.0 (0.2) | 0.1 (0.4) | 0.2 (0.5) | 0.1 (0.3) | 0.8 (2.0) | 0.8 (2.3) | 0.8 (2.0) |
| Productivity losses | 4.1 (4.7) | 3.5 (3.5) | 10.8 (9.6) | 12.0 (21.8) | 4.4 (5.6) | 24.9 (20.8) | 33.3 (28.7) | 26.3 (22.4) |
| **Total health system cost** | **1.0 (0.1)** | **2.8 (2.2)** | **8.4 (7.7)** | **11.0 (2.7)** | **2.6 (3.3)** | **43.8 (16.1)** | **47.1 (18.4)** | **44.3 (16.5)** |
| Drugs and medical supplies | 0.6 (0.1) | 2.1 (2.2) | 6.2 (7.1) | 7.6 (3.6) | 1.8 (2.9) | 34.7 (14.1) | 32.1 (15.4) | 34.3 (14.3) |
| Investigation cost | 0.3 (0.0) | 0.4 (0.3) | 1.6 (1.0) | 2.9 (2.6) | 0.5 (0.6) | 2.2 (2.2) | 6.2 (4.5) | 2.8 (3.1) |
| Routine services (visit/bed days) | 0.1 (0.0) | 0.4 (0.0) | 0.5 (0.0) | 0.5 (0.0) | 0.3 (0.1) | 6.9 (3.1) | 8.8 (1.9) | 7.2 (3.0) |
| **Total societal cost** | **6.3 (5.5)** | **9.5 (5.9)** | **30.4 (22.4)** | **31.6 (20.0)** | **10.0 (10.4)** | **85.9 (34.7)** | **108.2 (62.3)** | **89.5 (40.9)** |

iCCM: integrated community case management

**Table S5. Mean cost and standard deviation per outpatient and inpatient malaria episode by type of health facility and cost component from sensitivity analysis using multiple imputations (2023 United States dollars).**

| **Cost component** | **Outpatient** | | | | | **Inpatient** | | |
| --- | --- | --- | --- | --- | --- | --- | --- | --- |
|  | **iCCM (n**=153) | **Health center (n**=220) | **District hospital (n**=25) | **Regional hospital (n**=6) | **Overall (n=404)** | **District hospital (n**=186) | **Regional hospital (n=32)** | **Overall (n=218)** |
| **Total household cost** | **5.5 (5.4)** | **10.5 (57.1)** | **14.0 (15.1)** | **20.6 (21.0)** | **9.8 (50.4)** | **48.1 (67.8)** | **86.4 (63.0)** | **53.8 (68.4)** |
| Direct medical out-of-pocket expenses | 0.0 (0.1) | 0.1 (0.4) | 1.2 (2.4) | 2.6 (2.8) | 0.1 (0.7) | 2.4 (3.6) | 10.9 (10.3) | 3.6 (6.0) |
| Medications | 0.0 (0.1) | 0.1 (0.3) | 0.9 (2.0) | 2.6 (2.8) | 0.1 (0.6) | 2.2 (3.5) | 10.9 (10.3) | 3.5 (6.0) |
| Investigation | 0.0 (0.0) | 0.0 (0.1) | 0.3 (1.3) | 0.0 (0.0) | 0.0 (0.3) | 0.1 (0.4) | 0.0 (0.0) | 0.1 (0.4) |
| Consultation/services cost | 0.0 (0.0) | 0.0 (0.0) | 0.0 (0.0) | 0.0 (0.0) | 0.0 (0.0) | 0.1 (0.5) | 0.1 (0.5) | 0.1 (0.5) |
| Direct non-medical out-of-pocket expenses | 1.1 (1.9) | 4.0 (10.7) | 6.0 (5.9) | 5.9 (2.0) | 3.5 (9.6) | 15.6 (27.0) | 25.2 (20.5) | 17.0 (26.3) |
| Transportation | 0.3 (1.1) | 0.6 (1.4) | 2.7 (4.3) | 2.3 (1.0) | 0.6 (1.7) | 5.4 (14.5) | 6.6 (14.2) | 5.6 (14.4) |
| Meal | 0.6 (1.6) | 3.2 (10.6) | 3.3 (2.1) | 3.4 (1.8) | 2.8 (9.4) | 9.0 (22.3) | 16.4 (11.4) | 10.1 (21.2) |
| Accommodation | 0.0 (0.2) | 0.1 (0.4) | 0.0 (0.1) | 0.0 (0.1) | 0.1 (0.4) | 0.1 (0.6) | 0.0 (0.1) | 0.1 (0.5) |
| Other episode related expenses | 0.1 (0.4) | 0.1 (0.5) | 0.0 (0.3) | 0.2 (0.5) | 0.1 (0.5) | 1.0 (2.4) | 2.1 (2.9) | 1.2 (2.5) |
| Productivity losses | 4.5 (4.8) | 6.5 (56.0) | 6.7 (8.5) | 12.0 (21.8) | 6.1 (49.2) | 30.2 (61.5) | 50.3 (40.9) | 33.2 (59.3) |
| **Total health system cost** | **1.0 (0.1)** | **2.7 (2.9)** | **5.5 (6.5)** | **11.0 (2.7)** | **2.5 (3.1)** | **40.0 (21.6)** | **60.4 (31.2)** | **43.1 (24.4)** |
| Drugs and medical supplies | 0.6 (0.1) | 2.0 (2.9) | 4.0 (5.8) | 7.6 (3.6) | 1.8 (3.0) | 30.1 (20.4) | 41.5 (24.9) | 31.8 (21.5) |
| Investigation cost | 0.3 (0.0) | 0.3 (0.2) | 1.0 (1.0) | 2.9 (2.6) | 0.4 (0.4) | 2.4 (2.4) | 8.7 (6.8) | 3.3 (4.1) |
| Routine services (visit/bed days) | 0.1 (0.0) | 0.4 (0.0) | 0.5 (0.0) | 0.5 (0.0) | 0.3 (0.1) | 7.5 (4.6) | 10.2 (4.7) | 7.9 (4.7) |
| **Total societal cost** | **6.5 (5.5)** | **13.2 (57.3)** | **19.5 (20.6)** | **31.6 (20.0)** | **12.3 (50.6)** | **88.1 (72.4)** | **146.8 (86.1)** | **96.9 (77.4)** |

iCCM: integrated community case management

**Supplementary Table 6. Mean cost per malaria episode with standard deviations with gross national income (GNI)-based productivity losses estimates in 2023 United States dollars.**

| **Cost component** | **Outpatient** | | | | | **Inpatient** | | |
| --- | --- | --- | --- | --- | --- | --- | --- | --- |
|  | **iCCM (n=153)** | **Health center (n=220)** | **District hospital (n=25)** | **Regional hospital (n=6)** | **Overall (n=404)** | **District hospital (n=186)** | **Regional hospital (n=32)** | **Overall (n=218)** |
| Direct medical out-of-pocket expenses | 0.0 (0.1) | 0.2 (0.6) | 2.2 (2.9) | 2.6 (2.8) | 0.3 (1.1) | 2.6 (4.1) | 8.5 (12.5) | 3.5 (6.4) |
| Direct non-medical out-of-pocket expenses | 1.1 (2.1) | 3.0 (2.9) | 9.0 (6.5) | 5.9 (2.0) | 2.7 (3.5) | 14.5 (10.4) | 19.9 (15.1) | 15.3 (11.4) |
| Productivity losses | 2.4 (2.1) | 2.4 (2.5) | 3.1 (2.8) | 2.7 (2.2) | 2.4 (2.4) | 10.6 (6.8) | 14.8 (8.2) | 11.2 (7.2) |
| Total household cost | 3.6 (3.1) | 5.6 (4.6) | 14.3 (10.3) | 11.2 (3.5) | 5.4 (5.3) | 27.7 (16.8) | 43.2 (30.3) | 29.9 (20.1) |
| Total health system cost | 1.0 (0.1) | 2.8 (2.5) | 8.4 (7.7) | 11.0 (2.7) | 2.6 (3.4) | 42.8 (18.2) | 50.8 (23.3) | 44.0 (19.2) |
| **Total societal cost** | **4.6 (3.1)** | **8.4 (5.2)** | **22.7 (17.4)** | **22.2 (5.0)** | **8.0 (7.6)** | **70.5 (27.6)** | **94.0 (45.9)** | **73.9 (32.1)** |

iCCM: integrated community case management
